# Encounter with Huge Yolk Sac Ovarian Tumor in a Child: A Case Report

**DOI:** 10.31729/jnma.8041

**Published:** 2023-02-28

**Authors:** Suman Bikram Adhikari, Prabina Swornakar, Prabesh Paudel, Asmita Neupane

**Affiliations:** 1 Department of Pediatric Surgery, Grande International Hospital, Tokha, Kathmandu, Nepal; 2 Department of Emergency Medicine, Grande International Hospital, Tokha, Kathmandu, Nepal; 3 Kathmandu Medical College and Teaching Hospital, Sinamangal, Kathmandu, Nepal

**Keywords:** *children*, *surgical procedure*, *yolk sac tumour*

## Abstract

Yolk sac tumour frequently arises in the gonads as a type of germ cell tumour, though rare is a highly malignant ovarian tumour in children and prompt treatment should be done. We hereby report a case of malignant ovarian tumour presenting with an abdominal lump and increased urinary frequency. Different diagnostic modalities were used such as ultrasonography of the whole abdomen, contrast-enhanced computed tomography abdomen pelvis and tumour markers of betahuman chorionic gonadotropin and alpha-fetoprotein. This revealed an 18.2×14.3×10 cm mass likely a neoplastic germ cell tumour with minimal ascites. A tumour mass was found to arise from the left ovary and complete excision of the tumour along the left fallopian tube was done. Adjuvant chemotherapy started immediately. We hereby present a case of a 9-year-old girl with a huge yolk sac tumour of the left ovary which is rare in our setting and is presented here to differentiate any ovarian mass in this age group.

## INTRODUCTION

Ovarian masses can be solid or cystic with multivariate histology and are rare in the pediatric population. Incidence of ovarian tumours increases with age, from 0.43 in 100,000 cases at 1 year age to 152 in 100,000 cases in 35-year-old patients.^1^ Yolk sac tumour (YST) is the second most common tumour in malignant ovarian germ cell tumours.^2^ Diagnosis of YST is based on Ultrasonography (USG), computed tomography (CT), abdominal-pelvic magnetic resonance imaging (MRI) and histopathology. Tumour markers for YST are α-FP and β-HCG.^3,4^ Schiller-Duval body, pathognomonic for yolk sac tumours, appears like a glomerulus in structure with a fibrovascular core.^2^

## CASE REPORT

A 9-year-old girl was first presented to her general practitioner in local clinics with a history of fever with a maximum temperature of 38.33° C and was treated with some antibiotics on an outpatient basis. When the patient was assuming the supine position at her home, her mother noticed a bulge on her abdomen which upon palpation felt like a hard mass which was never talked about or examined. The patient was then brought to a tertiary care centre for further evaluation. The patient also complained of increased urinary frequency for 3-4 months, with no history of any vaginal bleeding or discharge. The patient's menarche had not started yet. The patient also complains of a recent loss of 3 kgs of weight in the past 3 months and night sweats a month prior. There was no history of any radiation exposure when she was conceived; the post-delivery status of the child was unremarkable. There was a history of bilateral ovarian cyst surgery in the mother of the patient. Furthermore, there is a history of ovarian cyst excision on the paternal side of the family (3 sisters of the father).

On physical examination, a palpable, firm, non-mobile hypogastric mass was observed. USG showed a huge well-defined heterogeneous solid cystic lesion measuring 17.1x12.2 cm in the pelvic cavity possibly arising from the left adnexal region showing internal vascularity, possibly neoplastic in nature.

**Figure 1:**
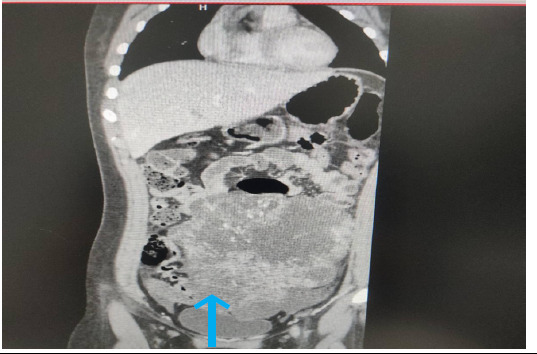
CT abdomen pelvis showing yolk sac tumour.

Computed tomography revealed a large solid cystic mass measuring approximately 18.2x14.3x10 cm in the pelvis, lower and central abdomen with no calcification or fat densities. Solid components showed intense heterogeneous enhancement in the post-contrast study and peripheral wall enhancement in cystic components. Mass supplied by the branches of internal iliac arteries and predominant drainage into the left gonadal vein which is enlarged measuring 10 mm. Mild indentation into the superior surface of the urinary bladder is seen with mild compression of both ureters with mild dilation of both pelvicalyceal systems. The left ovary is not separately visualized with the normal uterus and right ovary. Mildly enlarged aortocaval and para-aortic lymph nodes measuring 16x11 mm and 13x10 m respectively. Minimal free fluid is seen in the peritoneal cavity (Figure 1).

**Figure 2:**
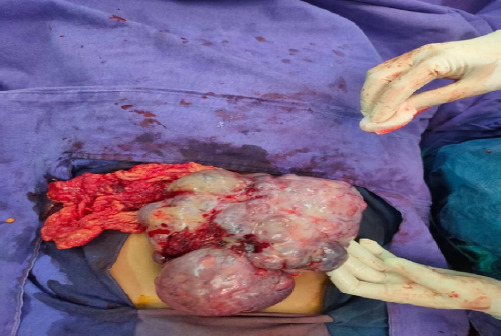
Intraoperative picture of an ovarian mass.

Features suggestive of malignant germ cell tumour arising from left ovary. The value of tumour markers, total beta-human chorionic gonadotropin and lactate dehydrogenase (LDH) were <2.39 mIU/ml and 417 U/l respectively and alpha-fetoprotein (AFP): 500 ng/ml. Thus, the patient underwent complete excision of the mass (weighing 3 kg and 40 g) along with the removal of the left fallopian tube (Figure 2).

A gross examination of the specimen showed no breach in the capsule and cut section showing solid (grey-white) and cystic (showing mucin in space) areas with areas of haemorrhage and necrosis. Hematoxylin and eosin-stained sections of the specimen revealed tumour cells arranged in nests, sheets, glands and microcystic patterns. Schiller-Duval bodies were also seen. The findings were consistent with YST without lymphovascular, ovarian surface or fallopian tube invasion.

The patient was discharged 4 days after the operation with unremarkable recovery. It was a staged I yolk sac tumour. The case was referred to a pediatric oncologist where she was advised to chemotherapy. The patient underwent four cycles of adjuvant chemotherapy and monitoring of chemotherapy effectiveness is done with an estimation of serum alpha-fetoprotein level.

## DISCUSSION

Yolk sac tumour which most frequently arises in the gonads as a type of germ cell tumour is rare in children but is highly malignant.^5^ Young patients with ovarian tumours often complain of acute or chronic abdominal pain, vomiting, nausea, increase of abdominal volume or other symptoms induced by the compression on surrounding organs.^2^ However, our patient also first presented with fever and abdominal bulge but later stated the fact that she had prior different symptoms due to compression of the surrounding organs like frequency of urination.

The imaging tools are extremely useful in establishing the diagnosis of ovarian tumours and they usually consist of pelvic and abdominal ultrasounds, CT scans and MRIs. Ultrasound is widely used in the assessment of multiple pediatric pathologies, such as abdominal tumours,^6^ and others too. In certain cases, abdominal-pelvic MRI may also be required. The ultrasound exam was very suggestive of the diagnosis in our case. As per other studies, AFP can be applied as a feasible tumour marker because its level was seen to be elevated in >90% of YST.^7^ In our case, AFP was found to be 500 ng/ml. The prognosis of patients can be monitored by the AFP level after the operation.^7^ It has been reported that the lowering level of post-operative serum AFP could be a useful marker for determining if residual cancer cells still exist after surgery. Serum alphafetoprotein has been used as a marker for the effect of postoperative radiation therapy and/or chemotherapy in cases of ovarian endodermal sinus tumours.^6^ AFP is specific in the yolk sac tumour, but it is not sensitive (overall sensitivity as low as 60%) because it can be seen in other cancers like hepatocellular carcinoma.

The general treatment for YST is surgery for eliminating the primary tumour without severe morbidity. The surgical approach of adnexal masses can consist of either laparotomy or laparoscopy depending on factors related to both the tumour and the patient.^10^

In our case, due to the huge size of the tumour, a laparotomy with complete excision was done and we did not encounter any post-surgical complications. The postoperative period was uneventful. Given the tumour size in our case, complete excision of the tumour along with a left fallopian tube and left ovary was done. For most of the patients with OGCT, unilateral scalping-oophorectomy with preservation of the contralateral ovary and the uterus was appropriate.^10^

As per guidelines, the present-day treatment for the yolk sac tumour is surgery and chemotherapy. Thus, the involvement of an oncologist is necessary. In the case of yolk sac tumours, most patients will need at least three cycles of chemotherapy. The bleomycin, etoposide and cisplatin (BEP) regimen is the best choice. A significant response to chemotherapy is indicated by a decrease in tumour marker levels following treatment. The diagnosis, the intervention, and the therapeutic plan must be based on the symptoms, pelvic ultrasound and potential serum markers. Furthermore, YST metastasizes through the hematogenous route in >50% of pediatric patients in comparison with only 4-6% of adult patients.^11^ After treatment, follow-ups are required such as abdominal and pelvic examination, CT, chest X-ray and AFP levels.

Proper presurgical investigations are of major importance because the Proper orientation of the therapeutic plan, the surgical approach, the surgical team, and the type of surgical intervention is only possible through proper presurgical investigations. With the aid of the paediatrician's and gynaecologist's communication abilities, a successful relationship with the patient and parents should be developed to get the best outcomes.

The clinical features of children with yolk sac tumours commonly present with abdominal pain and distension. However, our case presented initially with fever and later with abdominal distension and pressure symptoms due to the increasing size of the tumour. Management of the case was done by complete excision of the tumour along with the removal of the left fallopian tube and adjuvant chemotherapy. Due to the rarity of this tumour, it should be suspected in young children so that treatment can start early before metastasis. Prompt treatment and investigations should be started after the diagnosis of the tumour. A team effort is required for the best outcome in favour of the patient and parents.
